# Applications of Artificial Intelligence in Microbial Diagnosis

**DOI:** 10.7759/cureus.49366

**Published:** 2023-11-24

**Authors:** Yogendra P Shelke, Ankit K Badge, Nandkishor J Bankar

**Affiliations:** 1 Microbiology, Bhaktshreshtha Kamalakarpant Laxmanrao Walawalkar Rural Medical College, Ratnagiri, IND; 2 Microbiology, Datta Meghe Medical College, Datta Meghe Institute of Higher Education and Research (Deemed to Be University), Wardha, IND; 3 Microbiology, Jawaharlal Nehru Medical College, Datta Meghe Institute of Higher Education and Research (Deemed to Be University), Wardha, IND

**Keywords:** pathogen identification, epidemiological monitoring, antibiotic resistance prediction, artificial intelligence in healthcare, microbial diagnosis

## Abstract

The diagnosis is an important factor in healthcare care, and it is essential to identify microorganisms that cause infections and diseases. The application of artificial intelligence (AI) systems can improve disease management, drug development, antibiotic resistance prediction, and epidemiological monitoring in the field of microbial diagnosis. AI systems can quickly and accurately detect infections, including new and drug-resistant strains, and enable early detection of antibiotic resistance and improved diagnostic techniques. The application of AI in bacterial diagnosis focuses on the speed, precision, and identification of pathogens and the ability to predict antibiotic resistance.

## Introduction and background

Microbial diagnosis entails the identification of microorganisms through techniques such as culture, molecular analysis, and imaging, which constitute a pivotal domain within the realm of healthcare. It starts with appropriate sample collection and runs into several problems with conventional procedures, including sample handling, difficulty in culture, incorrect identification, and antimicrobial susceptibility testing difficulties. These traditional methods require manpower, and treatment is often delayed [1]. Artificial intelligence (AI) has revolutionized the field of microbial diagnostics by providing more precise and current findings. AI analyzes data, pattern recognition, and diagnostic processes faster. It is essential for early identification of the disease, advancement of treatment, custom treatment, and epidemic monitoring. Advanced data sets are analyzed by AI-driven algorithms to detect infections rapidly, anticipate disease outbreaks, and improve treatment approaches and outcomes [2]. The use of AI in microbial diagnosis raises concerns about ethics. Essential ethical considerations include protecting patient privacy, addressing algorithmic biases, maintaining data security, promoting transparency, and ensuring equal treatment. To maintain patient safety, personnel surveillance is still necessary [3]. As policies and ethical regulations for AI are implemented in healthcare, it can play a crucial role in providing precise, individualized diagnoses while ensuring patient rights and access to medical devices [4]. This review aims to analyze the use of AI in microbial diagnosis.

## Review

Microbial diagnosis

Identifying microorganisms through microbial diagnosis is vital to healthcare as it helps determine the causative agents of infections and diseases. The techniques employed for detection include cultivating bacteria and fungi on appropriate growth media, isolating viruses through cell culture, and characterizing the infectious agent through biochemical, antigenic, or genetic means [5]. Alternatively, infectious diseases may also be discerned by the detection of a specific immune response, typically in the form of antibodies that develop over the course of the illness. In some instances, the visualization of the infectious agent within infected tissue can furnish a diagnosis based on distinctive morphological attributes or, at the very least, aid in categorizing the type of organism responsible for the infection [6]. Conventional methodologies employed for this purpose are well established but recognized for their protracted timelines and demanding labor requirements. These conventional techniques encompass the utilization of culture media and labor-intensive biochemical tests. In contrast, contemporary approaches necessitate swift, cost-effective methodologies for the initial categorization of bacterial and fungal isolates. Moreover, modern analytical methods require comprehensive microbial profiling, often relying on molecular techniques, such as polymerase chain reaction (PCR), targeting the 16S ribosomal RNA gene. These findings are crucial in developing effective therapeutic strategies [1]. Diagnosing microorganisms involves multiple steps, beginning with sample collection, transport, and storage [7]. This is a crucial step in the diagnostic process, as the accuracy of subsequent test results depends on the proper collection and handling of clinical samples [8].

Challenges in traditional microbial diagnosis

Microbial diagnosis, a fundamental aspect of modern medicine and microbiology, faces challenges that span the entire diagnostic process. Sample collection and transport represent a critical initial challenge [9]. The process begins with the collection of clinical specimens. However, ensuring the correct and timely collection and transportation of clinical samples to the laboratory poses a formidable challenge. Improper handling, storage, and transport delays can increase the risk of the viability and integrity of microorganisms within the sample, leading to inaccurate diagnostic results [10]. Subsequently, the culture and isolation of microorganisms are considered a primary traditional diagnostic method. This process takes a long time for culture results. Improper cultivation of microbes raises the possibility of false negative results. The excessive use of antibiotics in clinical practice can further affect the growth and isolation of specific organisms [11].

Accurate identification and classification of microbial species represent another significant challenge. Traditional methods, including biochemical tests and microscopy, may not consistently detect the correct identification due to misleading morphological characteristics or atypical behaviors exhibited by certain microorganisms [12]. Determining the susceptibility of bacterial isolates to antibiotics in treatment selection is also challenging. Traditional susceptibility testing techniques, such as the disk diffusion method, are time-consuming and sometimes need to accurately predict the response of the infecting strain to antibiotics, leading to suboptimal treatment choices and potential therapeutic complications [13]. Traditional methods often require a team of highly qualified personnel, specialized equipment, and significant resources to diagnose medical conditions. There is an increased chance of human error in settings with limited resources due to the dependence on skilled professionals. As a result, it is crucial to develop new and user-friendly diagnostic methods [14].

Traditional microbial diagnosis often causes a delay in the initial phase of therapy, allowing diseases to progress uncontrolled due to a delay in results [15]. Empirical therapy increases antibiotic resistance, but traditional methods detect microorganisms, leading to misdiagnosing emerging pathogens [16]. Conventional diagnosis is fraught with the possibility of cross-contamination and false positives, making it more challenging. As traditionally performed, microbial diagnosis may not provide real-time data for effective epidemiological surveillance due to the risk of cross-contamination. This could lead to false-positive results from serological examinations [17]. The lack of standardized procedures and quality measures in diagnostic laboratories may result in inconsistent results that are less accurate and consistent [18]. Challenges in microbial diagnosis are depicted in Table 1.

**Table 1:** Challenges in microbial diagnosis by traditional methods.

Challenge/issue	Description
Sample collection and transport	Collection and transportation of clinical samples to the laboratory can be error-prone, affecting the viability and integrity of microorganisms and leading to inaccurate results [10].
Culture and isolation	Traditional culture methods are time-consuming, may yield false negatives, and can be impacted by the overuse of antibiotics [11].
Identification and classification	Correct identification of microbial species can be challenging due to the misleading morphological characteristics and atypical behaviors of certain microorganisms [12].
Antibiotic susceptibility testing	Traditional susceptibility testing techniques are slow and sometimes inaccurate, leading to suboptimal treatment choices and potential complications [13].
Dependence on skilled personnel	Traditional methods rely on highly qualified personnel, specialized equipment, and significant resources, making them error-prone in settings with limited resources [14].
Delay in initial therapy	Traditional microbial diagnosis can cause delays in treatment initiation, allowing diseases to progress and increasing the risk of antibiotic resistance [15].
Empirical therapy	The delay in diagnosis may lead to empirical therapy, which can promote antibiotic resistance and misdiagnose emerging pathogens [16].
Cross-contamination and false positives	Conventional methods carry a risk of cross-contamination and false-positive results, further complicating the diagnostic process [17].
Lack of real-time epidemiological surveillance	Traditional diagnosis may not provide real-time data for effective epidemiological surveillance due to the risk of cross-contamination and false positives [17].
Lack of standardized procedures and quality measures	Inconsistent results and accuracy issues may arise due to the absence of standardized procedures and quality measures in diagnostic laboratories [18].

The role of AI in microbial diagnosis

The application of AI in microbial identification and analysis has revolutionized the field, leading to more rapid and reliable results compared to conventional methods [19]. AI algorithms are capable of analyzing genomic data, aiding scientists and clinicians in identifying pathogens, predicting antibiotic resistance, and even discovering new microbial species. Furthermore, machine learning models can increase the speed and accuracy of microbial diagnosis by quickly analyzing complex data patterns [4]. The use of AI in microbe diagnosis has become a multifaceted and essential tool that encompasses pattern recognition, prediction modeling, and enhancement of microbe analysis efficiency. AI-powered algorithms have revolutionized the field by examining vast datasets of microbial information and identifying patterns and irregularities that would be challenging for human analysts to detect quickly and accurately. This capacity for pattern recognition is particularly critical in the early detection of infectious diseases, where rapid identification of viral species and their transmission patterns can inform effective containment strategies [2,4]. AI plays a crucial role in predictive modeling, using historical data to forecast microbial behavior and guide future decision-making. This predictive power is indispensable in foreseeing disease outbreaks, comprehending antibiotic resistance trends, and streamlining treatment protocols [20]. One of the best examples is that machine learning models, such as deoxyribonucleic acid (DNA) sequencer, can analyze the genomic sequences of bacteria and viruses to predict their propensity for mutations and resistance to specific drugs, guiding clinicians in selecting the most effective treatment options [21]. AI-powered automation speeds up processes such as sample processing, image analysis, and data interpretation. This not only shortens the diagnostic turnaround time but also allows healthcare professionals to concentrate on the most challenging aspects of patient care [19,22].

Early disease detection

Early Pathogen Detection

AI-powered algorithms have proven to be invaluable in quickly and effectively detecting microbial pathogens in clinical samples. These algorithms are trained to recognize specific patterns or genetic markers associated with various pathogens. This rapid and accurate detection of pathogens allows for immediate therapeutic interventions, significantly reducing the risk of infection growth [23].

Disease Outbreak Prediction and Hotspot Identification

AI plays a crucial role in predicting disease outbreaks and identifying hotspots. By analyzing a wealth of data sources, including medical records and environmental data, AI algorithms can identify trends and patterns indicative of potential outbreaks. This early detection enables public health authorities to implement targeted preventive measures and allocate resources where they are most needed [24].

Early Warning Systems

AI is integrated into early warning systems that continuously monitor data from diverse sources. These systems are designed to detect unusual patterns of microbial infections that may signal an emerging health threat. By scrutinizing data in real time, AI-driven early warning systems facilitate prompt investigation and intervention, helping to prevent the spread of diseases and mitigate their impact [25].

Real-Time Monitoring of Microbial Populations

The ability of AI to analyze real-time data is instrumental in monitoring microbial populations within healthcare facilities. By continuously tracking and analyzing changes in microbial populations, AI can promptly identify deviations from baseline patterns. This early detection capability allows for swift responses to potential health risks, such as hospital-acquired infections, ensuring patient safety and efficient healthcare delivery [25].

Drug discovery

AI-assisted microbial detection is changing not just clinical procedures but also the field of drug research to discover prospective therapeutic targets, improve the drug development process, and improve the search for antimicrobial agents to prevent a wide range of infections [26]. AI significantly affects the identification of targets since it analyzes microbial genomes, proteomes, and metabolic pathways. Machine learning models accelerate drug development by predicting affinities for compound microbial target binding, accelerating the selection of potential drugs for experimental validation [27]. AI can help improve drug repurposing by utilizing knowledge about authorized medications and their modes of action. By analyzing data, AI can identify recently approved medicines that can be repurposed for treating microbial diseases, potentially avoiding the time-consuming drug development process [28]. The impact of AI in predicting pharmacokinetic and pharmacodynamic characteristics is significant. AI-driven modeling reduces adverse effects, optimizes dosage, and ensures safety, effectiveness, and compatibility for clinical development [29]. To predict potential resistance mechanisms, AI algorithms analyze microbial genomic data. These data enable developers to create treatments that are less likely to cause resistance, promoting the development of more effective and durable antimicrobial drugs [4]. Various applications of AI in drug discovery and treatment are shown in Table 2.

**Table 2:** Application of AI in drug discovery and treatment. AI: Artificial intelligence.

Application	Description
Prospective therapeutic target identification	AI aids in the discovery of potential therapeutic targets by analyzing microbial genomes, proteomes, and metabolic pathways. This accelerates the drug development process by predicting affinities for compound-microbial target binding, expediting the selection of potential drugs for experimental validation [26,27].
Drug repurposing	AI leverages knowledge about authorized medications and their mechanisms of action to identify approved drugs that can be repurposed for treating microbial diseases. This approach can save time by avoiding lengthy drug development processes [28].
Pharmacokinetic and pharmacodynamic modeling	AI plays a significant role in predicting pharmacokinetic and pharmacodynamic characteristics of drugs. AI-driven modeling reduces adverse effects; optimizes dosages; and ensures safety, effectiveness, and compatibility for clinical development [29].
Predicting resistance mechanisms	AI algorithms analyze microbial genomic data to predict potential resistance mechanisms. This data-driven approach helps in developing treatments that are less likely to induce resistance, promoting the creation of more effective and durable antimicrobial drugs [4].

Treatment personalization

AI can offer innovative solutions for personalized treatment and epidemic monitoring. In microbial diagnosis, AI-powered algorithms have shown exceptional accuracy and efficiency in identifying pathogenic microorganisms from various clinical samples. These algorithms analyze large datasets of genetic, proteomic, and clinical information to diagnose infections quickly and precisely, allowing healthcare providers to develop tailored treatment plans [30]. The ability to rapidly detect and analyze patterns in epidemiological data has significantly improved epidemic monitoring. By processing and interpreting real-time data from various sources, such as social media, healthcare records, and environmental sensors, AI systems can recognize emerging outbreaks, track the spread of diseases, and predict potential hotspots. This proactive approach allows for timely intervention measures, facilitating containment and mitigation efforts during infectious disease crises [31]. In essence, AI in microbial diagnosis and epidemic monitoring not only advances healthcare by optimizing treatment strategies but also plays a pivotal role in safeguarding public health on a global scale [2].

Epidemic monitoring

AI has proven to be an effective tool in the monitoring of epidemics. Its wide-ranging applications in this field are transformative and multifaceted. Additionally, by analyzing various factors, including climate, population density, and travel patterns, machine learning models may predict disease breakout trends that promote health authorities with the development of prevention intervention techniques [32]. In real time, AI can analyze clinical and epidemiological data to aid contact tracking and assess the effectiveness of containment measures. AI-assisted microbial detection is revolutionizing the identification and management of epidemics, ultimately saving lives and decreasing the impact of infectious diseases on global health [33]. Table 3 presents the advantages and disadvantages of AI in microbial diagnosis [2,15,19,30,34,35].

**Table 3:** Advantages and disadvantages of AI in microbial diagnosis. AI: Artificial intelligence.

Advantages	Disadvantages
Rapid analysis of microbiological data enables fast diagnosis, improving accuracy and minimizing false positives for better patient results.	AI systems may be costly to purchase and install, which could pose difficulties for smaller healthcare facilities and those with limited finances.
AI systems can identify intricate details and patterns that humans may miss, leading to more accurate identification of pathogens and their characteristics.	AI learning and training require unbiased, high-quality data, as incomplete or biased data may lead to incorrect diagnoses and treatment suggestions.
AI can assist academics in making justifiable decisions by identifying hidden patterns for valuable scientific discoveries.	AI decision-making processes can be difficult to understand, which may lead healthcare professionals to distrust AI recommendations.
Personalized treatments utilizing patient data can enhance outcomes and minimize adverse effects.	AI systems may provide inaccurate suggestions due to the biases they acquire from the training data.
AI reduces manual labor requirements, resulting in labor savings on expenses. Diagnosis may be lower in cost when it relies less on costly laboratory equipment.	Qualified experts are necessary for monitoring AI operations and analyzing results. Highly qualified AI experts are abundant in certain regions.
AI systems are constantly evolving to stay ahead of novel diseases.	Healthcare practitioners are refusing to adopt AI due to job displacement fear.
Remote deployment of AI-powered diagnostics increases access to healthcare, reaching populations with limited resources and spanning geographic barriers. AI is particularly useful in times of epidemics.	Ethical and privacy issues arise in the handling of patient data used for diagnosis purposes.
AI minimizes human errors, resulting in consistent and verifiable outcomes, making treatments safer and more successful.	The lack of internet access in resource-limited regions may hinder the application and maintenance of AI systems.

Ethical considerations

The integration of AI into microbial diagnosis is a rapid advancement that presents unparalleled opportunities to improve healthcare outcomes. Healthcare data can be gathered and stored online by social media, genetic testing, and bioinformatic companies. These data can be hacked and used for unethical purposes [36]. These ethical issues encompass a wide range of topics, including patient privacy, algorithmic biases, data security, accessibility, and overall responsibility of healthcare providers [37].

Patient Privacy and Informed Consent

Ensuring patient privacy is one of the primary ethical concerns of using AI systems. Since these systems rely on vast datasets that may contain sensitive health information, it is crucial to protect patient privacy [38]. Ethical guidelines require informed consent from patients before collecting and using their data for AI-driven diagnostics. It is essential to balance anonymizing data to protect patient identities and retaining clinical relevance to ensure ethical use of patient information [39].

Algorithmic Biases and Fairness

AI may be associated with structural barriers like language and cultural differences or situational challenges and may influence the inclusion of a particular patient in the data. Automated customer service or scheduling software may cause systematic disparities. Then, natural language processing may be associated with gender and racial biases. Consequently, it is essential that developers and healthcare organizations put robust procedures for bias recognition and management [40].

Data Security and Cybersecurity

There are new data security concerns due to the increased reliance on connected systems and cloud-based data storage. Patient data must be protected against cyberattacks, and unauthorized access is ethically required. Healthcare organizations and AI developers must invest in adequate cybersecurity protections, encryption procedures, and compliance with privacy regulations to protect the confidentiality and integrity of patient information [41].

Transparency

Clinicians and patients must understand the way AI-powered diagnostic decisions work. The advancement of transparent AI models that can provide quick justifications for their predictions is encouraged by ethical standards. As a result, AI systems are more trusted, and healthcare professionals can confidently act on AI advice [42].

Accessibility and Equity

It is morally required to ensure appropriate access to AI-powered diagnostic tools. Healthcare organizations should work to close the technological gap and give underprivileged communities access to innovative AI diagnosis. To promote integration and appropriate access to healthcare, AI-driven healthcare solutions must additionally keep people with disabilities [3].

Human Oversight and Accountability

AI has the potential to improve the accuracy of diagnoses, but it should continue the role of healthcare personnel. Maintaining a balance between AI-driven automation and human intelligence is necessary for ethical practice and patient safety. Healthcare providers are responsible for patient care. AI should be viewed as a tool that supports and improves medical opinion rather than replacing it [19].

The future of AI in microbial diagnosis

AI-powered diagnostic tools will have a significant impact as machine learning and computational capabilities improve. The development of AI systems has transformed disease management and public health responses by quickly identifying infections, including novel and drug-resistant strains. Individuals will eventually be able to monitor their health and identify microbial diseases in real time [43]. The role in vaccine development, antimicrobial resistance, and epidemiological surveillance will continue to expand, enhancing our ability to respond to infectious diseases effectively [44]. As ethical considerations and regulatory frameworks continue to develop, the use of AI in microbial diagnosis is expected to become a vital component in the significant transformation of the future of healthcare by providing personalized and accurate diagnostic solutions, ensuring patient privacy protection and equitable access to innovative healthcare technologies [45].

## Conclusions

The technique for microbial diagnosis is evolving as an outcome of the integration of AI. It enables rapid and accurate pathogen detection, early detection of antibiotic resistance, and improved diagnostic techniques. Additionally, AI is essential for early diagnosis of diseases, drug development, personalized treatment, and outbreak detection. As a result, public health benefits and healthcare outcomes have improved considerably. However, as AI becomes more active in medical decision-making, ethical concerns such as patient confidentiality, algorithmic biases, data security, transparency, accessibility, and human supervision must be addressed. Even further improvements in disease treatment and prevention are acceptable in the future with the advancement of AI in microbial diagnosis, which also ensures ethical and just healthcare practices.
